# Inflammation-Induced Reactivation of the Ranavirus Frog Virus 3 in Asymptomatic *Xenopus laevis*

**DOI:** 10.1371/journal.pone.0112904

**Published:** 2014-11-12

**Authors:** Jacques Robert, Leon Grayfer, Eva-Stina Edholm, Brian Ward, Francisco De Jesús Andino

**Affiliations:** Department of Microbiology and Immunology, University of Rochester Medical Center, Rochester, United States of America; University of Colorado, Boulder, United States of America

## Abstract

Natural infections of ectothermic vertebrates by ranaviruses (RV, family *Iridoviridae*) are rapidly increasing, with an alarming expansion of RV tropism and resulting die-offs of numerous animal populations. Notably, infection studies of the amphibian *Xenopus laevis* with the ranavirus *Frog Virus 3* (*FV3*) have revealed that although the adult frog immune system is efficient at controlling RV infections, residual quiescent virus can be detected in mononuclear phagocytes of otherwise asymptomatic animals following the resolution of RV infections. It is noteworthy that macrophage-lineage cells are now believed to be a critical element in the RV infection strategy. In the present work, we report that inflammation induced by peritoneal injection of heat-killed bacteria in asymptomatic frogs one month after infection with *FV3* resulted in viral reactivation including detectable viral DNA and viral gene expression in otherwise asymptomatic frogs. *FV3* reactivation was most prominently detected in kidneys and in peritoneal HAM56+ mononuclear phagocytes. Notably, unlike adult frogs that typically clear primary *FV3* infections, a proportion of the animals succumbed to the reactivated *FV3* infection, indicating that previous exposure does not provide protection against subsequent reactivation in these animals.

## Introduction

Infections and die-offs caused by ranaviruses (RVs, family *Iridoviridae*) are increasing in prevalence concomitant with an unprecedented and alarming rise in the numbers of susceptible host species (including amphibians, bony fishes and reptiles) [1], [2]. Notably, the remarkable ability of RVs to cross species barriers of numerous ectothermic vertebrates, suggests that these pathogens possess potent immune evasion mechanisms [3]. Furthermore, although some ectothermic vertebrate species are highly susceptible to RVs, others are more resistant and may serve as asymptomatic carriers that disseminate infectious virus. While RV infections and host pathogen interactions are increasingly documented for a variety of amphibian species, there is still very little known about mechanisms of ranaviral pathogenicity and host immune defenses to these large complex DNA viruses, whose genomes encode some 100 genes [4]–[6]. Using *X. laevis* and the RV *Frog Virus 3* (*FV3*), we have established a reliable experimental system to elucidate at the cellular and molecular levels interactions between RV pathogens and their amphibian hosts [7], [8]. While our studies have shown the critical roles of CD8 T cells and antibody responses in controlling *FV3* infections, our findings also underscore a prominent role for macrophage-lineage cells both in the defense against RVs and as contributors to their immune evasion and possibly persistence [9].

In mammals, macrophage cell populations consist of long-lived, terminally differentiated and extensively heterogeneous immune cell populations, indispensable to host immunity and homeostasis. As sentinels of the immune system, macrophages recognize viral infections through a repertoire of pattern recognition receptors, and facilitate viral clearance by producing an array of bioactive molecules, as well as by serving as antigen presenting cells able to activate T cells [10]. Conversely, distinct macrophage subsets may become productively infected by certain viruses (e.g., human immunodeficiency virus [HIV], measles, etc.), and serve as long-term viral reservoirs and agents of pathogen dissemination [11]–[13]. However, the viral strategy for utilizing macrophage lineages for immune escape, persistence and spread within their hosts has thus far been documented primarily for RNA viruses [13]. Notably, during the early stages of *FV3* infections, there is an accumulation of activated macrophages in the peritoneal cavity, exhibiting increased pro-inflammatory cytokine gene expression (IL-1β and TNFα) [14], [15]. Intriguingly, subsequent to the resolution of the infection, peritoneal macrophages isolated from some, but not all asymptomatic animals harbor transcriptionally inactive *FV3* [14], [15]. Based on these findings we have hypothesized that although adult frog macrophages are integral to anti-*FV3* immune responses, some of these cells are permissive to this pathogen and harbor quiescent, non-replicating virus. Similar to mammals, *Xenopus* peritoneal leukocytes (PLs) are a heterogeneous group of cells that, based on morphology and Giemsa staining patterns, include polymorphonuclear (PMN) granulocytes as well as monocytes and macrophage-like cells, and smaller lymphocytes [16], [17]. We previously demonstrated that peritoneal injections of *X. laevis* with heat-killed *Escherichia coli* results in the accumulation of large numbers of PLs primarily composed (70 to 80%) of macrophages [18]. The present study examines the capacity to reactivate quiescent *FV3* in previously infected, asymptomatic *X. laevis* by inflammatory stimulation of these animals through intra-peritoneal administration of heat-killed *E. coli*.

## Material and Methods

### Animals

Outbred adult frogs were obtained from our *X. laevis* research resource for immunology at the University of Rochester (http://www.urmc.rochester.edu/smd/mbi/xenopus/index.htm). For all experiments outbred 2 year-old (∼6 cm length) adult frogs were infected by intraperitoneal (i.p.) injection of 1×10^6^ pfu of *FV3* in 0.2 ml of PBS modified to amphibian osmolarity (APBS) using a 1 ml sterile syringe with a 22 gauge, 1½ inch needle. Animals were maintained individually in 1 L container and reared by standard husbandry (feeding, cleaning). Virally infected animals were euthanized to minimize suffering as soon as abnormal behavior (listlessness, altered swimming and feeding, etc.) or signs of acute systemic infection (edema, floating a the surface) was detected or they displayed. Euthanasia was done by immersion in a 0.5% aqueous solution of tricaine methane sulfonate (MS-222), buffered with sodium bicarbonate.

### Ethics Statement

Experiments involving frogs were carried out according to the Animal Welfare Act from the United States Department of Agriculture (USDA), the Public Health Service Policy (A-3292-01) and the Public Health Act of New York State. Any discomfort was minimized at all times. Animal care and all the protocols has been reviewed and approved by the University of Rochester Committee on Animal Resources (Approval number 100577/2003-151).

### Frog Virus 3 Stocks and Animal Infections

Fathead minnow cells (FHM; American Type Culture Collection, ATCC No. CCL-42) were maintained in Dulbecco's modified Eagle's medium (DMEM; Invitrogen) supplemented with 10% fetal bovine serum (FBS; Invitrogen), penicillin (100 U/mL) and streptomycin (100 µg/mL) with 5% CO_2_ at 30°C. *FV3* was grown by a single passage on FMH cells, purified by ultracentrifugation on a 30% sucrose cushion and quantified by plaque assay on FMH monolayer under an overlay of growth media containing 1% methylcellulose [14]. A6 kidney cells (ATCC No. CCL-102) were maintained in the same DMEM culture medium diluted to amphibian osmolarity (addition of 30% water).

### Bacterial Stimulation

*E. coli* (XL1-blue, Stratagene, La Jolla, Ca.) cultured overnight at 37°C, were boiled for 1 hour, pelleted by centrifugation and resuspended in 1/10 of the initial volume (approximately 1×10^8^ bacteria/ml) of APBS [19]. Infected frogs were injected i.p. with 300 µl of the heat-killed bacteria mixture (3×10^7^ bacteria; corresponding to 3 mg of protein).

### Plaque Forming Assays

*FV3*-infected kidneys, PLs or A6 cells were lysed in hypotonic buffer (Tris-HCL 50 mM; pH 7.5) by 3 freezing/thawing cycles and serially diluted in DMEM supplemented with 2.5% FBS. Five hundred µl of each dilution was plated in duplicate onto confluent monolayers of FHM cells in 6-well plates, and incubated at 30°C for 1 h with gentle agitation of volumes of viral particles every 20 min. Remaining volumes of viral particles were removed by aspiration and 3 ml of overlay medium (DMEM supplemented with 2.5% FBS and 1% methyl-cellulose (Sigma) was added. Cells were incubated for 7 days at 30°C in 5% CO_2_. Overlay media was aspirated and the cells stained for 10 min with 1% crystal violet in 20% ethanol.

### PCR, RT-PCR and Quantitative Real-time PCR (qPCR)

RNA and DNA were extracted from cells and tissues using Trizol reagent following the manufacturer's protocol (Invitrogen). Total RNA (0.5 µg in in 20 µl) was used to synthesize cDNA with the iScript cDNA synthesis kit (Bio-Rad, Hercules, CA). One µl of cDNA template was used in all RT-PCRs and 50 ng DNA for PCR. Minus reverse transcriptase (RT) controls were included for every reaction, and all primers spanned at least one intron (Table 1). A water-only control was included in each reaction. PCR products were separated on 1.0% agarose gels and stained with ethidium bromide. Sizes of the products were determined using standardized markers of 1 kb plus from Invitrogen (Carlsbad, CA).

**Table 1:** Primer sequences used in this study.

Primers	Sequences
GAPDH	F: 5′ - ACCCCTTCATCGACTTGGAC - 3′
	R: 5′ - GGAGCCAGACAGTTTGTAGTG - 3′
EF-1α	F: 5′ - CCTGAATCACCCAGGCCAGATTGGTG - 3′
	R: 5′ - GAGGGTAGTGTGAGAAGCTCTCCACG - 3′
*FV3* DNA Poly II	F: 5′ - ACGAGCCCGACGAAGACTACATAG - 3′
	R: 5′ - TGGTGGTCCTCAGCATCCTTTG - 3′
MCP	F: 5′ - GACTTGGCCACTTATGAC - 3′
	R: 5′ - GTCTCTGGAGAAGAAGAAGAA - 3′
MCSF-R	F: 5′ - TGTATTCTTTGG ACT TGC CGT ATCTGG - 3′
	R: 5′ - TTGTTTAGCTTCAAATTCTGGGTAATA - 3′

a F, forward; R, reverse.

### Cytospins and Cell Staining

Peritoneal leukocytes (PLs; 2×10^5^ cells in 200 µl volume) were cytocentrifuged using a Shandon Southern cytospin centrifuge (600 rpm, 5 min.), fixed with 3.7% formalin for 1 min, permeabilized with 100% cold methanol (−20°C) and briefly washed with APBS. After blocking with 1% BSA in APBS for 1 hr, the PLs were incubated overnight with rabbit anti-*FV3* 53R serum [20] and mouse anti-HAM56 mAbs [21]. After washing, cells were incubated with DyLight 488-conjugated F(ab')2 Donkey Anti-Rabbit IgG (H+L) (Jackson ImmunoReaserch, PA) or DyLight 594-conjugated F(ab')2 Donkey Anti-Mouse IgG (H+L) (Jackson ImmunoResearch, PA), respectively. Cells then were stained with the fluorescent DNA intercalator (Hoechst-33258). Preparations were mounted in anti-fade medium (Molecular Probes, Oregon) and visualized with a Leica DMIRB inverted fluorescence microscope with a cooled charge-couple device (Cooke) controlled by Image-Pro software (Media Cybernetics).

### Statistics

One-way ANOVA and Long-rank (Mantel Cox) comparisons tests were performed using GraphPad Prism version 6.00 for Windows, GraphPad Software, La Jolla California USA, (URL: www.graphpad.com).

## Results

### 1. Detection of *FV3* DNA and transcripts in peritoneal leukocytes upon bacterial stimulation of previously challenged, asymptomatic adults

**Figure 1:**
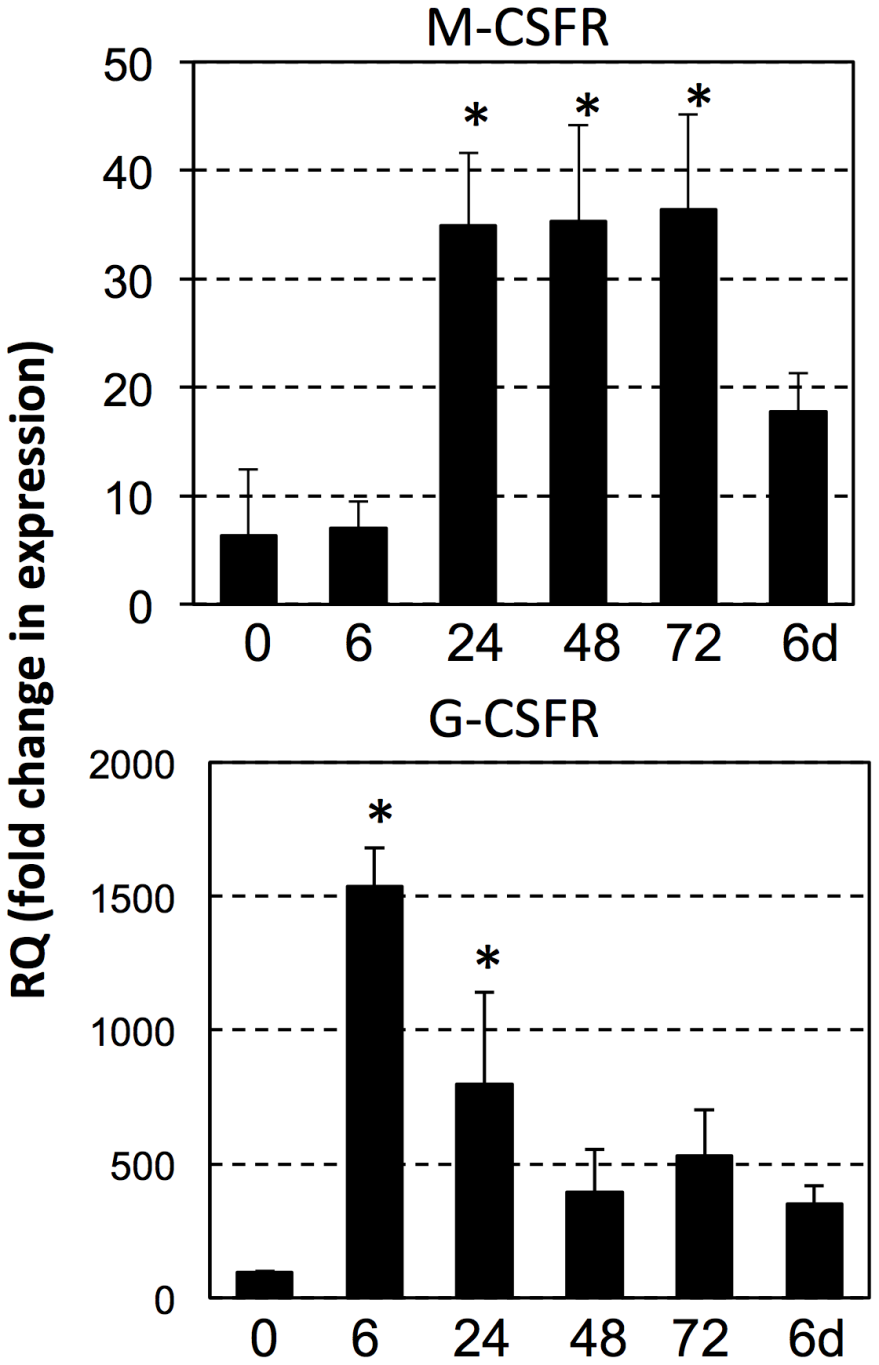
Change in gene expression of the macrophage marker M-CSFR and the granulocyte marker G-CSFR following injection of heat-killed bacteria. At different time points (6, 24, 48, 72 hrs and 6 days) following injection of heat killed *E. coli*, peritoneal phagocytes were isolated and assessed by qRT-PCR for genes expression of M-CFSR and G-CSFR. Gene expression was normalized relative to the GAPDH control. Results are means ± SEM; (*) over bars indicates significant differences between untreated control and experimental animals (3 individual per group).

Intraperitoneal injection of heat-killed (HK) bacteria (*E. coli*) into *X. laevis* adults results in a robust recruitment and accumulation of leukocytes in the peritoneal cavity within 3 days [14]. Based on Giemsa staining and morphological analysis, these elicited peritoneal leukocytes (PLs) were primarily composed of mononuclear phagocytes (>80%). To confirm these observations, we assessed the changes in the gene expression of macrophage and granulocyte growth factor receptors (M-CSFR and G-CSFR, respectively) during HK *E. coli*-elicited PL accumulation. Whereas the G-CSFR transcript levels showed an early transitory increase at 6 hrs following bacterial stimulation and declined to baseline levels by 24 hrs, the M-CSFR gene expression markedly increased from 24 hrs to 72 hrs post-stimulation (Fig. 1; corresponding to experiment 3 in Table 2). These data are in good concordance to a predominant accumulation of mononuclear phagocytes 3 days after bacterial stimulation and further support our previous observations [14].

**Table 2:** Detection of viral DNA and viral transcription in *X. laevis* adult's PL cells lysates after *FV3* infection or *FV3* + Bacterial stimulation.

Treatment a	Experiments	Total Number of frogs	PCR b	RT-PCR b	Sign of infection	Death c
***FV3*** ** + ** ***E. coli***	Exp. 1	10	7	7	2	3 (30%)
	Exp. 2	10	5	5	4	5 (50%)
	Exp. 3	15	11	11	5	7 (43%)
	Exp. 4	10	5	2	2	1 (10%)
	**Total**	**45**	**28**	**25**	**13**	**16 (36%)**
***FV3*** ** only**	Exp. 1	10	3	3	ND	0 (0%)
	Exp. 2	10	1	0	ND	0 (0%)
	Exp. 3	20	2	3	3	4 (20%)
	Exp. 4	10	3	0	ND	0 (0%)
	**Total**	**50**	**9**	**6**	**3**	**4 (8%)**
***E. Coli*** ** only**	Exp. 1	10	ND	ND	1	1 (10%)
	Exp. 2	10	ND	ND	ND	1 (10%)
	Exp. 3	13	ND	ND	ND	0 (0%)
	**Total**	**33**	**ND**	**ND**	**1**	**2 (6%)**
**APBS**	Exp. 1	5	ND	ND	ND	0 (0%)
	Exp. 2	5	ND	ND	ND	0 (0%)
	Exp. 3	8	ND	ND	ND	0 (0%)
	**Total**	**18**	**ND**	**ND**	**ND**	**0 (0%)**

a Treatments: Adult frogs were treated by i.p. injection with: APBS only (30 days treatment); *E. coli* only (3 days treatment); *FV3* only (30 days treatment); or *FV3* + *E. coli* (30 days treatment with *FV3* preceding of 2 days of recovery and 3 days treatment with HK *E. coli*).

b FV3 detection by the indicated method; ND: not detected.

c Death that resulted from the treatments. All frogs in each group were monitored and death was recorded daily for 60 days after each treatment.

**Figure 2:**
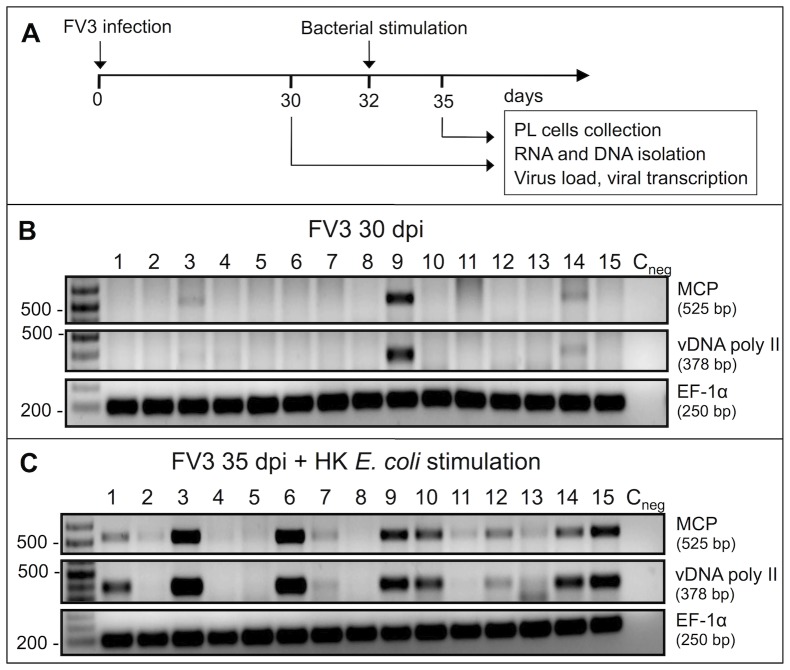
Bacterial stimulation-mediated reappearance of *FV3* DNA in PLs of adult frogs after viral clearance. (**A**) Experimental outline. (**B**) PCR assay on total DNA purified from PLs harvested from 15 different outbred frogs (Experiment 3 in Table 2) at 30 dpi, and (C) PCR assay on DNA purified from PLs collected from the same animals 3 days after bacterial stimulation (35 dpi). Presence of viral DNA was assed by PCR on 50 ng of total DNA using *FV3* specific primers for MCP and vDNA poly II as well as *X. laevis* Ef-1α as a loading control.

We next assessed whether HK *E. coli* stimulation of adult frogs with asymptomatic *FV3* infection (i.e., usually exhibiting undetectable levels of *FV3* DNA after immune clearance) may result in recruitment of macrophages harboring quiescent *FV3* from the periphery into the peritoneum and the reactivation of this quiescent virus. To this end, two-year old adult frogs were infected with a sub-lethal dose (10^6^ PFU) of *FV3* and maintained for 30 days to ensure viral clearance, which usually occurs within 2 weeks [9]. At 30 days post-infection (dpi), the efficacy of viral clearance was evaluated by PCR and RT-PCR analysis of PLs. Subsequently (at 32 dpi), animals were injected with HK *E. coli* and PLs were again isolated 3 days later (35 dpi; Fig. 2A). Following these procedures, frogs were maintained under observation for further 60 more days (until 90 dpi total).

Initially, we examined whether viral DNA could be detected by PCR in PLs prior to bacterial stimulation, using *FV3* major capsid protein (MCP) and the viral DNA polymerase II-specific primers (vDNA pol II; *FV3* ORF 60R [22]). Before the inflammatory stimulus, detectable levels of *FV3* DNA were seen in 3 out of 15 frogs (1 prominently, and 2 at the limit of detection with this assay), indicating effective viral clearance at 30 dpi (Fig. 2B). In stark contrast, *FV3* DNA was detected in the PLs of 12 out 15 individuals 3 days after bacterial stimulation (35 dpi; Fig. 2C). Furthermore, the high intensity of some of the detected PCR products was suggestive of extensive viral loads (Fig. 1C; lane 3 and 6). As summarized in Table 2, comparable findings were obtained from 4 independent experiments. On average, 62% (28/45) of bacterially-stimulated frogs exhibited *FV3* reactivation (35 dpi), compared to only 6% seen in these animals just before the administration of the inflammatory stimulus (30 dpi). *FV3* was not detected in animal whole blood or purified blood leukocyte fractions (Fig. S1). This is consistent with previous observations indicating that *FV3* is only detected in blood circulation during early systemic infections [8], [23].

**Figure 3:**
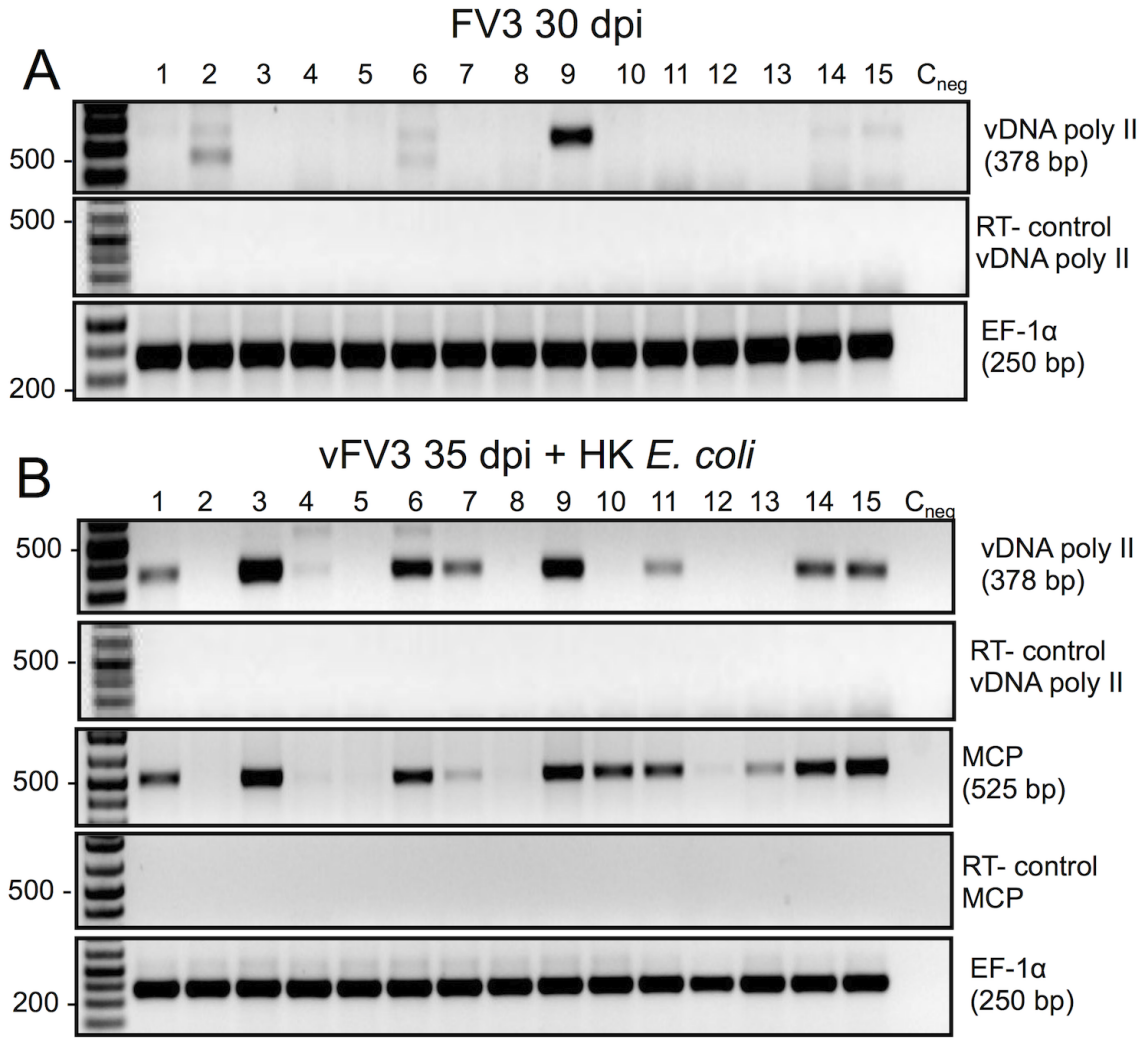
Detection of viral gene expression in PLs of frogs following bacterial stimulation. RT-PCR assay of DNase-treated RNA purified from PLs harvested from the same 15 different frogs used in Fig. 2 ((Experiment 3 in Table 2) at 30 dpi (A), and 3 days later after bacterial stimulation at 35 dpi (B) using *FV3* specific primers for vDNA pol and MCP as well as *X. laevis* Ef-1α as a loading control. RT minus controls were included to rule out contamination by genomic DNA.

To confirm that the inflammatory stimuli resulted in active infections, we examined *FV3* gene expression at 35 dpi (3 days post bacterial stimulation) of two integral viral genes: the early-expressed vDNA Pol II critical for RV replication and the late-expressed major capsid protein (MCP) that encodes the RV capsid subunits. RT-PCR analysis of cDNA synthesized from DNAse-treated RNA sample (Fig. 3) revealed that the expression of these *FV3* genes closely reflected the presence or absence of the *FV3* DNA (Fig. 2; Table 2). PLs from 8 of 15 HK *E. coli*-stimulated individuals showed significant FV3 gene expression (Fig. 3). Notably, similar results were obtained in the 3 additional experiments, whereas there was no detectable viral gene expression in cDNA samples mock-synthesized in absence of the reverse transcriptase (-RT), ruling out possible viral genomic DNA contamination of the RNA/cDNA samples (Fig. 3).

We conclude that the residual persisting *FV3* in a fraction of asymptomatic *X. laevis* adults may reestablish infections following bacterial stimulation. In addition, *FV3* gene expression suggests that FV3 detected in PLs after bacterial stimulation is transcriptionally active.

### 2. Reactivation of quiescent *FV3* by bacterial stimulation results in increased frog host mortality

**Figure 4:**
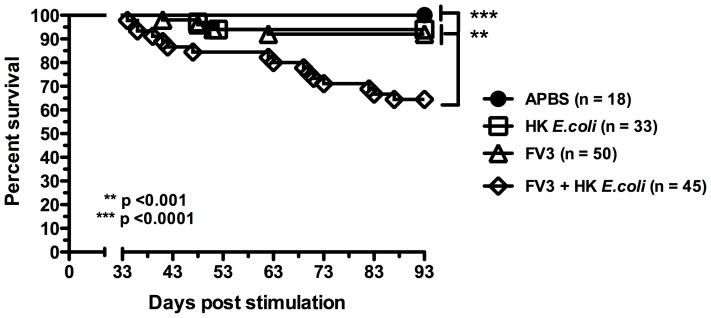
Survival of asymptomatic *FV3* infected adult frogs following bacterial stimulation. Kaplan-Meier curves represent outbred individual animals from three different experiments (35 total animals) that were infected with 1×10^6^ PFU of *FV3*, subjected to a peritoneal lavage at 30 dpi, injected with heat-killed bacteria and subjected to another peritoneal lavage at 35 dpi, then monitored daily for signs of *FV3* infection and death for 60 days (*FV3*+HK *E. coli*). Control animals included a group of 18 uninfected animals injected with saline vehicle (APBS), a group of 33 animals shame-infected with APBS that underwent peritoneal lavages and bacterial stimulation (HK *E. coli*), and a group of 40 animals infected with 1×10^6^ *FV3* (FV3). Statistical analysis was performed using the log-rank test (Mantel Cox) of a GraphPad Prism version 6.00 for Windows, La Jolla California USA, (URL: www.graphpad.com). The result are as follows: APBS vs HK *E. coli*, P<0.1; APBS vs *FV3*, P<0.1; APBS vs *FV3*+HK *E coli*, P<0.001; HK *E. coli* P vs *FV3*+HK *E. coli*, P<0.001.

To determine whether the inflammatory stimuli-induced *FV3* reactivation results in productive viral infections, we monitored animal survival over a period of 2 months. Strikingly, a significant fraction of these animals exhibited symptoms of acute ranaviral infection (Table 2) and ultimately succumbed from systemic viral infection (Fig. 4). It is noteworthy that *X. laevis* adult frogs are typically resistant to primary *FV3* infections [8]. Indeed, initial *FV3* inoculation of a control group from the same animal cohort resulted in a 10% mortality rate (see below and Table 2), which is consistent with our previous published observations [8], [23]. In addition, only two animals died from bacterial stimulation alone (6%), likely reflecting complications from anesthesia. In contrast, the induction of systemic lethal infections was consistently obtained in several independent experiments and reached 36% animal mortality within a period of 60 days following bacterial stimulation (90 dpi total).

**Figure 5:**
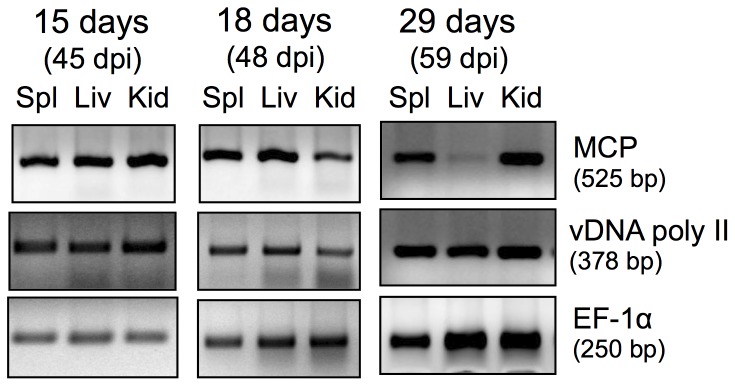
PCR diagnostic of asymptomatic *FV3* infected adult frogs that died following bacterial stimulation. PCR was performed on DNA extracted from spleen (Spl), liver (Liv) and kidneys (Kid) of a representative of frogs that died 15, 18 or 29 days after bacterial stimulation (45, 48 or 59 dpi, respectively) using primers specific for *FV3* MCP, vDNA poly II and EF-1α as control.

We additionally scored these animals for hallmark symptoms of RV disease including lethargy, skin redness (hemorrhage), shedding and edema [23]. Hemorrhage and tissue damages in the kidney and liver were also confirmed during necropsy of animals that died or were euthanized because of severe signs of sickness. We verified the occurrence of systemic *FV3* infection by PCR assays, and substantial viral DNA was detected in most tissues including spleens, livers and kidneys (Fig. 5).

Whereas *FV3* DNA was detected by PCR in most of the frogs that subsequently succumbed from FV3 infections, a few animals with undetectable viral loads in PLs following bacterial stimulation developed subsequent systemic *FV3* infections. It is possible that in these cases *FV3*-harboring macrophages (and/or other cells) were not recruited into the peritoneum or alternatively that the kinetics of reactivation in these animals were slower (than 33 dpi). Conversely, several frogs with detectable *FV3* DNA and viral gene transcripts did not develop systemic infection, suggesting that in these instances the host immune systems were effectively controlling the RV reactivated infections.

### 3. Detection of *FV3*-infected peritoneal macrophages after reactivation by bacterial stimulation

**Figure 6:**
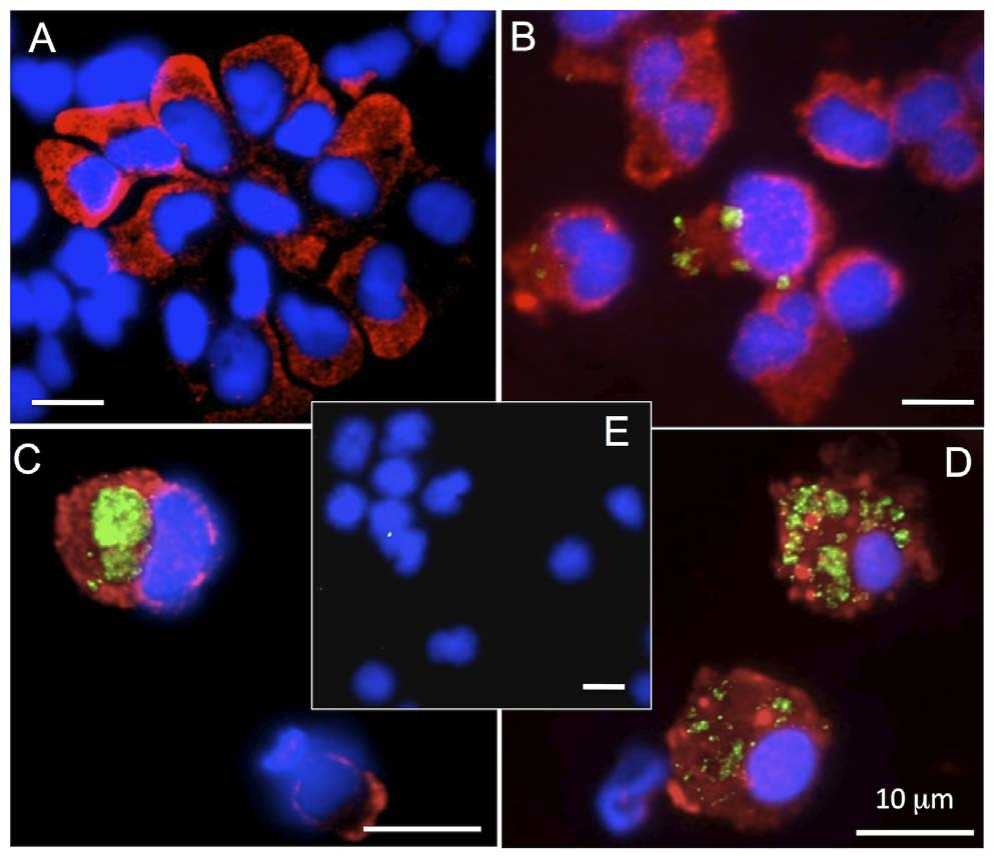
Visualization by immunofluorescence of infected peritoneal monocytic leukocytes from asymptomatic *FV3* infected adult frogs following bacterial stimulation. PLs were harvested from uninfected controls (A, E) or asymptomatic *FV3* infected outbred animals 3 days after bacterial stimulation (B, C D). Cells were cytocentrifuged on microscope slides, fixed with formaldehyde, permeabilized with ethanol, then stained with a rabbit anti-53R and Dylight 488-conjugated donkey anti-rabbit Abs (green) followed by anti-HAM56 mAb and Dylight 594-conjugated anti mouse Ab (red). Cells were then stained with the DNA dye Hoechst-33258 (Blue) mounted in anti-fade medium and visualized with a Leica DMIRB inverted fluorescence microscope. Bar represents 10 µm in each panel. (A) PLs from a bacterially stimulated, non-*FV3* infected frog stained with anti-HAM mAb; (B) PLs with low level of 53 specific signal from reactivated asymptomatic *FV3* infected animals; (C, D) PLs with high levels of 53 specific signal from reactivated asymptomatic *FV3* infected animals; (E) PLs from a bacterially stimulated, non-*FV3* infected frog stained with anti-53R Ab.

We have previously demonstrated that *X. laevis* peritoneal phagocytes harbor apparently quiescent *FV3* subsequent to general immune clearance of the viral infections [14], [15]. We hypothesize that *FV3* could then persists in these macrophages, and serve as reservoir for viral dissemination upon appropriate re-stimulation. We were therefore interested to determine whether *FV3* infected cells can be detected within mononuclear or polymorphonuclear peritoneal phagocyte populations. Accordingly, we examined PLs by fluorescence microscopy using the anti-53R Ab to detect *FV3* infected cells [20] and the macrophage marker HAM56 [21]. As we previously observed [14], [18], PLs harvested from non-*FV3*-infected *X. laevis* 3 days after bacterial stimulation were mainly composed of macrophages, staining positive for HAM56 (Fig. 6A). More importantly, the anti-53R Ab positively stained a substantial fraction (from 20% to as much as 60% for the heavily infected animals) of peritoneal macrophages from animals with reactivated *FV3* infection. Indeed, these 53R+ mononuclear phagocytes not only stained positive for HAM56 but also exhibited typical macrophage morphology (e.g., reniform nucleus, scattered granulation, ruffled membranes). Similar to epithelial cells infected by *FV3* [23], the cellular 53R-staining pattern of these macrophages was strictly cytosolic with punctate structures of different sizes (Fig. 6B–D). Whereas some macrophages exhibited faint positive 53R signal (Fig. 6B), others were strongly stained for 53R (Fig. 6C, D). In addition, there were no obvious signs of apoptosis (e.g., pyknotic nuclei) or marked cellular damage in the infected macrophage cells (Fig. 6 and data not shown). Intriguingly, no HAM negative cells were stained with the anti-53R Ab., indicating that *FV3* peritoneal reactivation was confined to HAM+ mononuclear phagocytes. Finally, no cells were stained by the anti-53R in PLs from uninfected, bacterially stimulated controls (Fig. 6E).

### 4. Inefficiency of peritoneal macrophages to produce infectious virus

**Figure 7:**
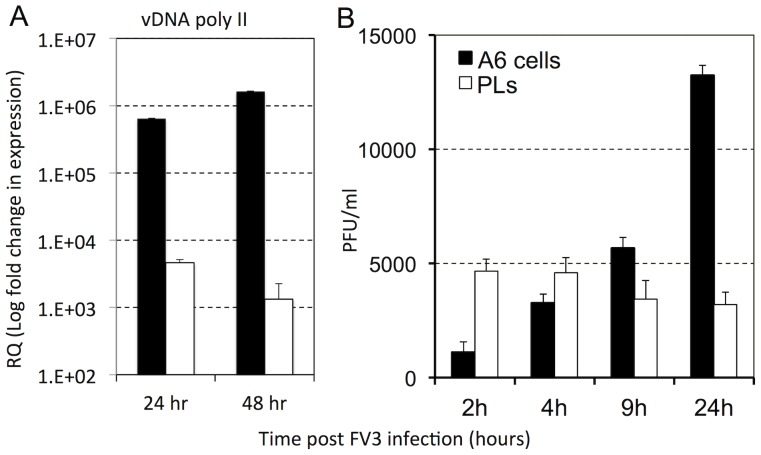
Comparison of viral gene expression and infectious virus produced by PLS and A6 kidney cell line infected in vitro with *FV3*. PLs from uninfected outbred animals and *Xenopus* A6 kidney cells were seeded at 1×10^6^ cells/well and infected with a MOI of 1 for (A) 24 and 48 hrs or (B) 2, 4, 9, 24 hrs. (A) The PLs and A6 cultures infected for 24 and 48 hrs were assessed for *FV3* vDNA Pol II gene expression by the delta∧deltaCT method qRT-PCR, using GAPDH as an endogenous control. (B) The *FV3* infectious burdens were enumerated by performing plaque assays on *FV3*-infected PLs and A6 culture lysates. Results are means ± SEM, *N* = 3.

Given our previous [14], [15] and present (Fig. 6) observations indicating that *FV3* infection of macrophages is different from that of epithelial cells such as in the kidney, we investigated the ability of PLs to support *in vitro* viral replication and to produce infectious virus. For this purpose, we infected in parallel at an MOI of 1 equal numbers (1×10^6^) of PLs obtained from uninfected bacterially elicited frogs and the *X. laevis* A6 kidney cell line (Fig. 7). To establish that the cells were infected, they were assayed for *FV3* vDNA Pol II gene expression as in Fig. 3. Whereas vDNA Pol II gene expression was detected in both cell types, indicating that both were infected, there were approximately 2 logs greater viral gene expression from the A6 cells at 24 h and 3 logs by 48 h as compared to PLs (Fig. 7A). Furthermore, plaque assay analysis revealed that *FV3* infection of PLs resulted in little to no virus production, whereas a significant increase in *FV3* production was seen in the A6 cells by 24 h (Fig. 7B). Notably, PL lysis and cell death remained negligible for 6–7 days (20–30%) after infection, whereas most A6 cells were lysed within this time frame (data not shown).

**Figure 8:**
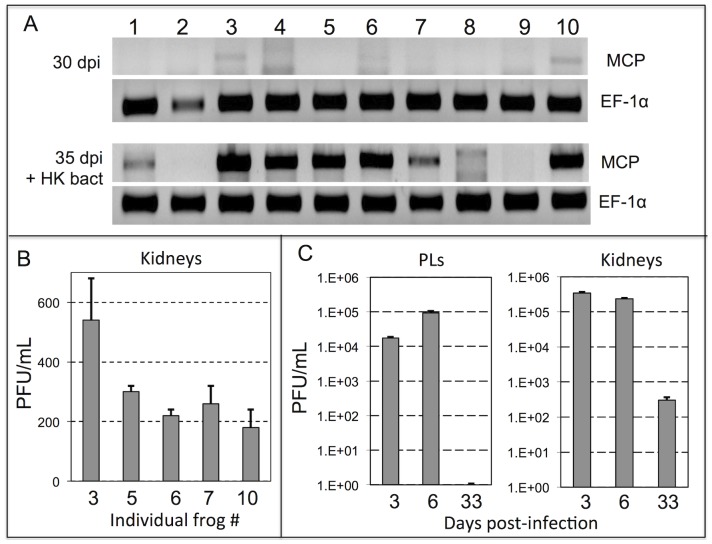
Detection of infectious virus in kidneys but not PLs of reactivated asymptomatic infection. (A) PCR assay on total DNA purified from PLs harvested from 10 different outbred frogs at 30 dpi and 35 dpi, (3 days after bacterial stimulation). Presence of viral DNA was assed by PCR on 50 ng of total DNA using *FV3* specific primers for MCP as well as *X. laevis* Ef-1α as a loading control. (B) Number of infectious *FV3* (PFU/ml/whole kidney) detected in kidney by plaque assay for frogs number (#) 3, 5, 6, 7, 10 from A sacrificed at 35 dpi. (C) Number of infectious *FV3* detected by plaque assay from PLs and kidneys of *FV3* infected frogs at 3, 6 and 33 dpi (3 individual for each time point).

Owing to the apparent lack of *FV3* expansion in *X. laevis* PL cultures *in vitro* (Fig. 7), we next assessed whether virus was produced during HK *E. coli*-mediated *FV3* reactivation *in vivo*. PLs and kidneys from *FV3* infected and reactivated animals were harvested and assessed (Fig. 8). Consistently, a significant fraction of these animals exhibited *FV3* reactivation in PLs as determined by PCR (Fig. 8A). However, plaque assays revealed a complete absence of infectious viral particles within these cells. In contrast, kidney tissues from five randomly selected animals yielded notable infectious *FV3* particles (Fig. 8B). To confirm the inability of PLs to foster infectious *FV3 in vivo*, another pool of adult frogs was infected for 3, 6 and 30 days before PLs and kidneys were harvested for plaque assay (Fig. 8C). Notably, infectious virus was detectable in PLs harvested at 3 and 6 dpi but not at 35 dpi following inflammatory stimulation (Fig. 8C). In contrast, the kidney tissues contained significant PFU of *FV3* during the primary infection (3 & 6 dpi) and following reactivation with HK *E. coli*, 33 dpi (Fig. 8C).

These data further substantiates the poor ability of *FV3* harboring PLs to produce infectious virus progeny, suggesting that the kidney may also be the primary site of productive infection upon FV3 reactivation.

## Discussion

This study represents the first report detailing concrete evidence of the roles of macrophage-lineage cells in the persistence of RV infections in otherwise resistant and asymptomatic hosts. Furthermore, our findings pertaining to the capacity of inflammatory stimuli such as bacterial challenge to reactivate and reestablish previously dormant ranavirus infections, often to the detriment of the amphibian hosts, reflect a previously unappreciated mechanism of RV dissemination.

Although macrophages have been found infected by RVs in amphibian [24], [25] and reptile [26], [27] species, little is known about the precise roles of these cells during RV infections. As in mammals, we have shown that *Xenopus* macrophages are actively involved in early host antiviral innate immune responses [14], and serve as antigen presenting cells able to activate T cells [28], [29]. Interestingly, studies using *FV3* inoculations of rats as a hepatitis model have underlined the preferential tropism of *FV3* for liver macrophages (Kupffer cells, [30]). Notably, shortly after infection viral particles have been detected in phagocytic vacuoles and endocytic compartments of these macrophages [31]. Although mammalian cells grow at 37°C and are thus not permissive to ranaviral replication, these earlier studies suggest that mononuclear phagocytes represent cellular targets of RV infections, presumably due to their high phagocytic and endocytic activities. Our present work using the *Xenopus* animal model supports the notion that *FV3* infection of peritoneal macrophages is distinct from epithelial cell infiltration by this virus. In mammals, certain RNA viruses such as HIV or measles infect macrophage subsets to evade immunity and persist in a latent or quiescent state within their hosts [11], [12]. However, this strategy is not typical of large DNA viruses such as *FV3*. Poxviruses such as vaccinia, which have similarities to RVs and are thought to be phylogenetically related [32], are not known to remain quiescent in host cells, but have evolved a number of genes targeting multiple host immune mechanisms including macrophage responses [33]. Herpesviruses like HSV-1 targets fibroblast for latency, whereas Epstein Barr virus uses B cells [34]. Our data showing the preponderant role of macrophages in long term quiescent infection of *Xenopus* by one of the RV main type *FV3* suggests that RVs have evolved new mechanisms to persist in their hosts.

We previously reported that *FV3* DNA was detected in a significant fraction of apparently healthy *X. laevis* adults obtained from various suppliers, suggesting that such asymptomatic carriers could serve as vectors of RV dissemination in the wild [15]. Presumably, physiological and/or immune perturbations may trigger productive reactivation of quiescent *FV3*, leading to high viral titer shedding. This is exemplified in the present study where an inflammatory stimulus in the form of HK *E. coli* resulted in prominent re-activation of *FV3* infection. Unlike LPS, which is not readily immuno-stimulatory to *Xenopus* [18], [35], heat-killed bacterial stimulation within frog peritonea elicits robust accumulation of macrophages that display multiple features of activation including increased expression of inflammatory genes (Il-1β, TNFα), stress proteins (gp96) and scavenger receptors (CD91) [18], [36]. Our gene expression data suggest that as in mammals, *X. laevis* coordinate the inflammatory recruitment of granulocytes, followed by macrophages, represented by marked PL expression increases of G-CSFR then the M-CSFR, respectively. The present study also suggests that heat-killed bacteria may serve not only as stimulus of *Xenopus* macrophage recruitment and activation, but may also facilitate *FV3* reactivation within a fraction of these cells. At present, the abundance and tissue distribution of *FV3* harboring macrophages following viral clearance is unclear, although our past work indicates that these cells may reside in the peritoneum and/or the kidney [14], [23]. It is noteworthy that in our previous [14], [15] and present studies we have failed to detect *FV3* in polymorphonuclear peritoneal leukocytes. This is intriguing considering the related myelopoietic origins of granulocytes and macrophages. However, granulocytes are typically short-lived and thus may represent poor viral reservoirs, whereas some macrophage subsets exhibiting uncanny longevity would be ideal for viral persistence.

Our present findings as well as a recent related work [37] converge to indicate that *X. laevis* macrophages effectively harbor *FV3*, but do not facilitate viral expansion or extensive progeny virus production. This may well be advantageous to the virus, where in absence of high intracellular titers, *FV3* may better evade host immune detection, avoid cell stress and activation, and otherwise disseminate and remain quiescent within its amphibian hosts. Presumably, under appropriate conditions, for example when a secondary infection would skew the immune response towards anti-bacterial rather than anti-viral immunity, *FV3* may leave its macrophage hiding place and re-infect its primary tropic cells, such as the kidney epithelia (as seen here).

Presumably, bacterial stimulation increases the proportion of macrophages harboring *FV3* through peritoneal recruitment/accumulation, while concomitantly it elicits *FV3* reactivation. The precise mechanism involved in these processes remains to be elucidated. However, it is remarkable that the reactivation of these chronic quiescent RV infections appears to circumvent immune defenses, especially considering that *Xenopus* adults are inherently resistant to *FV3* and develop efficient immunological memory, resulting in faster and more potent viral clearance upon acute re-infection [8], [9]. This implies that the route of *FV3* reactivation subverts the immunological barriers that established during the primary infection. Presumably, upon *FV3* re-challenge of previously infected animals with exogenous virus, the virus now encounters the established and expanded adaptive immune components, rapidly controlling and clearing the reinfection. Conversely, it is reasonable that RVs have evolved to bypass this established adaptive barrier from within the organism through the establishment of quiescence in subsets of immune (macrophage) cells and efficient reactivation following appropriate stimuli such as an immune response skewed away from viral clearance.

Our findings have relevance for RV infection of other amphibian species, considering the variable susceptibility observed across anurans, especially adult animals [7]. In the face of the amphibian declines, it is crucial to consider that resistant amphibian species with persistent asymptomatic RV infections may represent major, previously overlooked facets of RV dissemination. Indeed, in addition to co-infections of amphibians by RVs and other pathogens such as bacteria, fungi and parasites, chronic inflammation may also be induced by a variety of physiological stressors including habitat perturbation, resource competition and pollution. These various effects may well be compounding towards quiescent RV reactivation in wild amphibian populations. As such, the remarkable capacity of *FV3* and presumably other RVs to chronically persist in migrating reservoir hosts represent an additional avenue for infection dissemination; likely contributing to the rapid expansion of these pathogens and possibly the escalating amphibian decline.

## Supporting Information

Figure S1 **Detection of infectious virus in kidneys but not PLs of reactivated asymptomatic infection.** PCR assay on total DNA purified from PBLs and erythrocytes from blood collected from 15 different frogs at 30 dpi, and from the same animals 3 days after bacterial stimulation (35 dpi). Presence of viral DNA was assed by PCR on 50 ng of total DNA using *FV3* specific primers for MCP and vDNA poly II as well as *X. laevis* Ef-1α as a loading control.
(PDF)
