# Supplementary material for: Inflammation-Induced Reactivation of the Ranavirus Frog Virus 3 in Asymptomatic Xenopus laevis
Source: PLoS One. 2014 Nov 12;9(11):e112904. doi: 10.1371/journal.pone.0112904 (PMC4229299; doi:10.1371/journal.pone.0112904)
Supplement: Figure S1 — Detection of infectious virus in kidneys but not PLs of reactivated asymptomatic infection. PCR assay on total DNA purified from PBLs and erythrocytes from blood collected from 15 different frogs at 30 dpi, and from the same animals 3 days after bacterial stimulation (35 dpi). Presence of viral DNA was assed by PCR on 50 ng of total DNA using FV3 specific primers for MCP and vDNA poly II as well as X. laevis Ef-1α as a loading control. (PDF) [file pone.0112904.s001.pdf]

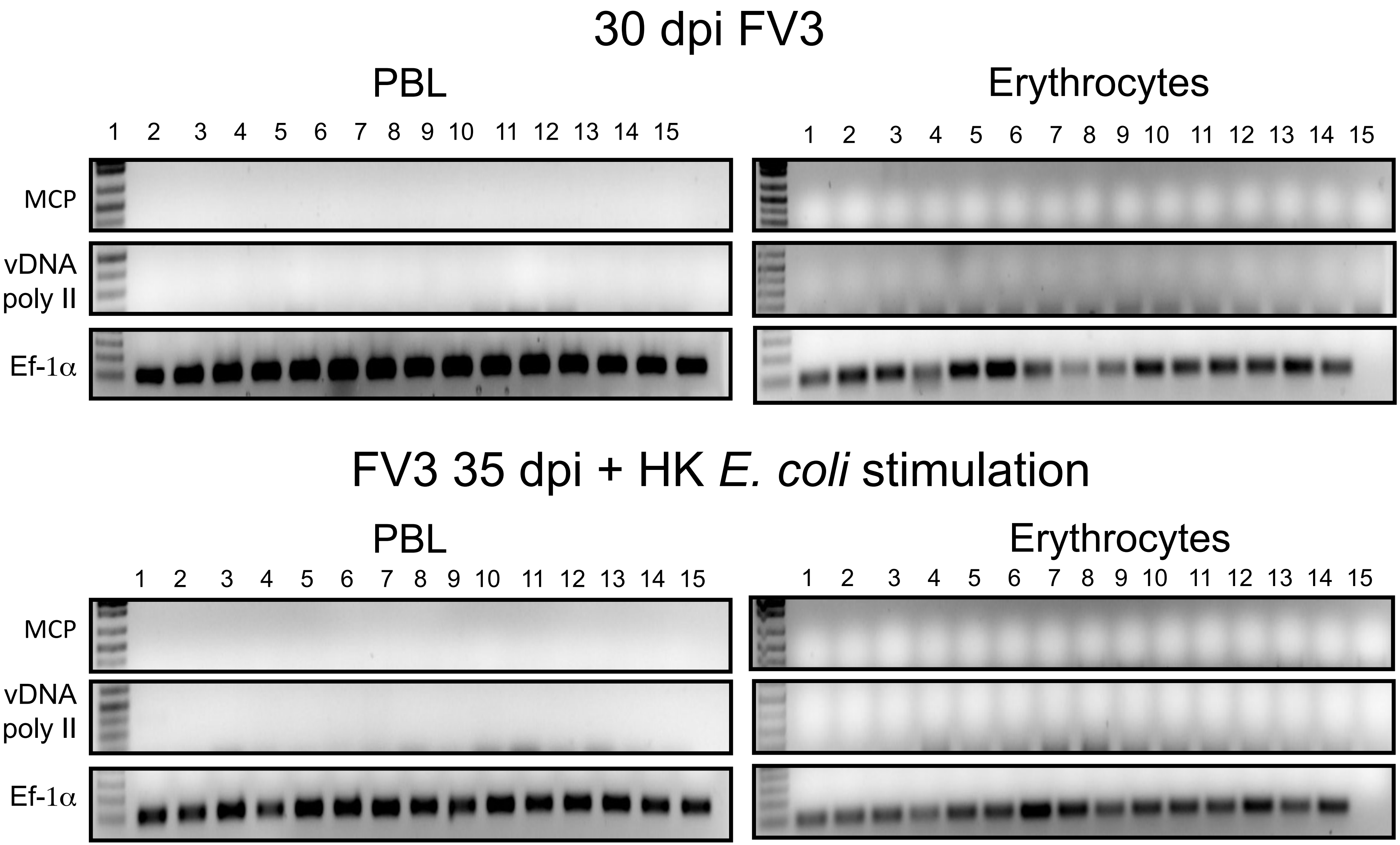

Supplementary Fig. 1: Lack of detectable viruse in PBLs and erythrocytes from frogs with reactivated asymptomatic infections. PCR assay on total DNA purified from PBLs and erythrocytes from blood collected from 15 different frogs of experiment 3 (Fig. 1; Table 1) at 30 dpi, and from the same animals 3 days after bacterial stimulation (35 dpi). Presence of viral DNA was assed by PCR on 50 ng of total DNA using FV3 specific primers for MCP and vDNA poly II as well as *X. laevis* Ef-1a as a loading control.
